# Incorporating the video communication assessment for error disclosure in residency curricula: a mixed methods study of faculty perceptions

**DOI:** 10.3389/frhs.2025.1503922

**Published:** 2025-08-29

**Authors:** Emily Grossniklaus, Ann M. King, Angelo E. D’Addario, Karen Berg Brigham, Thomas H. Gallagher, Thea G. Musselman, Kendra Hester, Kathleen M. Mazor, Andrew A. White

**Affiliations:** ^1^ Department of Medicine, VA Puget Sound Healthcare System and University of Washington School of Medicine, Seattle, WA, United States; ^2^ National Board of Medical Examiners, Philadelphia, PA, United States; ^3^ Collaborative for Accountability and Improvement, University of Washington School of Medicine, Seattle, WA, United States; ^4^ Department of Medicine and Department of Bioethics, University of Washington School of Medicine, Seattle, WA, United States; ^5^ Department of Medicine, UMass Chan Medical School, Worcester, MA, United States; ^6^ Department of Medicine, University of Washington School of Medicine, Seattle, WA, United States

**Keywords:** graduate medical education, medical error disclosure, mixed methods, formative assessment, curriculum change, communication skills assessment, faculty perceptions, crowdsourcing

## Abstract

**Introduction:**

U.S. resident physicians are required to demonstrate competency in disclosing patient safety events to patients, including harmful errors. The Video-based Communication Assessment (VCA) is a novel tool that provides opportunities to practice and receive feedback on communication skills. VCA practice and feedback are associated with improvements in residents’ error disclosure skills, but no research exists regarding faculty members’ views on implementing the VCA in patient safety curricula. We sought to evaluate faculty members’ views on using the VCA for teaching error disclosure communication in residency, and to identify barriers and facilitators to VCA adoption.

**Methods:**

Mixed methods study using a validated survey of Acceptability, Appropriateness, and Feasibility (AAF), and thematic content analysis of structured key informant interviews with faculty.

**Results:**

25 faculty completed both the AAF survey and interview. Overall, the faculty rated the VCA with a mean AAF score of 4.23 (out of 5). Analysis of the interviews identified case quality, relevancy, and fulfillment of a curricular void as attractive aspects of the tool, while feedback delays and content were identified as limitations. A major challenge to implementation included finding curricular time. Faculty anticipated the VCA would be useful for resident remediation and could be used in faculty coaching on error disclosure.

**Conclusion:**

The VCA seems to be an acceptable and feasible tool for teaching error disclosure; this finding warrants confirmation and testing in other specialties. Faculty members expected the VCA would be useful for both improving poor performance as well as informing faculty coaching, although these approaches remain untested. To facilitate adoption, faculty recommended protecting curricular time for VCA use and effectively communicating with residents about who will review their personal assessments and how the exercise will support their learning.

## Introduction

After harmful medical errors, physicians often fail to provide the information and emotional support sought by patients (1, 2). This has serious consequences; patients suffer and lose trust in healthcare, physicians often experience significant distress, and institutions are more likely to face lawsuits (2, 3). Communication and Resolution Programs (CRPs) try to help patients after they are harmed, but may fall short because many doctors still find error disclosure to be challenging (4–6). Physicians cite inadequate training as a key contributor to their difficulty with these conversations (7). To address this gap, the Accreditation Council for Graduate Medical Education (ACGME) recently introduced stringent residency program requirements that “residents must demonstrate competence in using tools and techniques that promote patient safety and disclosure of patient safety events” (8). Unfortunately, the teaching methods currently in widespread use may fall short (9). Lectures and on-line modules do not reliably impart communication skills, and informal learning in the clinical environment often lacks feedback from supervisors and patients (10–12). Although practice with standardized patients may facilitate skill development, it is rarely used, costly, unscalable, difficult to standardize, and prone to statistically unreliable assessments (13–15). Emerging alternatives, including virtual communication assessments, may offer an opportunity for feedback-rich skill development (16–18). To reach ACGME goals and make CRPs work, educators need simple tools they can add to patient safety curricula.

The Video-based Communication Assessment (VCA) lets trainees practice their error disclosure skills and receive clear feedback, all on a phone or computer (19). The VCA presents video vignettes of simulated cases and directs users to audio-record a spoken response to the patient in the scenario. The responses are reviewed by crowdsourced panels of laypeople, who provide ratings on key domains of error disclosure quality on a five-point scale and qualitative responses on what they would have ideally liked the users to have said. Users receive feedback reports consisting of an assessment summary rating, exemplary responses, and “learning points” intended to provide actionable general guidance derived from raters' comments (20, 21). VCA ratings from laypeople have proven reliable and consistent with rating of patients who have been harmed by care (22, 23).

In a recent multisite randomized trial using the VCA, self-directed review of crowdsourced feedback was associated with higher ratings of residents' error disclosure skills (24). Despite these encouraging findings, its feasibility and acceptability for widespread adoption in residency training is unknown. Key questions include whether the VCA is appropriate for patient safety curricula, what features may support or impede its integration, and what enhancements would facilitate its uptake by both residents and faculty. To address these questions, we conducted a mixed-methods study exploring internal medicine and family medicine residency faculty members' thoughts on the VCA as a tool to teach error disclosure communication skills in their institutions, including barriers to adoption and ways the tool might be improved. Answering these questions could accelerate adoption of a proven technique for preparing residents for effectively fulfilling a key physician role in CRP execution.

## Materials and methods

### Trial of a novel approach to teaching error disclosure skills

This study of faculty experiences and beliefs took place in the context of a pragmatic educational trial, the results of which were previously published (24). We briefly summarize the trial design and intervention here. To evaluate the effectiveness of VCA feedback in resident error disclosure training, we conducted a multisite randomized trial in 2022–23 with 146 post-graduate year 2 (PGY2) residents in Internal Medicine (IM) and Family Medicine (FM). Residents received a lecture about error disclosure over Zoom from an investigator (AAW or THG), completed two VCA cases at Time 1, and were randomized to control or the intervention (crowdsourced feedback provided in the VCA app within 2 weeks for self-directed review). Residents then completed a second VCA exercise after 4 weeks (Time 2). The study found that self-directed review of crowdsourced feedback was associated with higher ratings of residents’ error disclosure skill. This manuscript describes the beliefs of residency faculty about the intervention (VCA use and crowdsourced feedback to residents) and its suitability for wider adoption.

### Participants, setting, VCA practice

Participants included internal medicine and family medicine faculty who teach patient safety and disclosure techniques at seven U.S. academic centers (list as follows): University of Washington (WA), University of Washington at Boise (ID), Washington State University (WA), Beaumont Health (MI), Dartmouth (NH), University of Massachusetts (MA), and Washington University (MO). All sites had participating IM programs, and two had FM program participation in addition to IM. Each program director was asked to identify at least four faculty (including core faculty, program directors, and assistant/associate program directors) responsible for training residents about medical error disclosure, who would be approached for recruitment. Up to three emails invitations were sent to eligible faculty. Faculty were paid a $60 incentive for completing the study; this amount was reviewed by the University of Washington IRB and deemed not to cause undue influence to participate.

To develop familiarity with the VCA, participants first used the VCA app to practice simulated error disclosure with the same medical error cases given to the residents in the randomized trial. They also completed a brief demographic survey within the VCA app, with questions about age, gender, race, specialty, and years in practice. After receiving personal feedback in the app in the same fashion as residents, they were invited to complete a survey and a research interview. We did not study the recordings or ratings of faculty VCA responses.

### Acceptability, appropriateness, and feasibility survey procedures and analysis

We assessed views on integrating the VCA into residency training using a widely used, reliable, brief, valid, 12-item tool (25). The tool has a 5-point scale ranging from *completely disagree* (1) to *completely agree* (5), with three subscales for Acceptability, Appropriateness, and Feasibility. Participants were also asked about their experience with coaching and instructing residents about error disclosure. The survey was coded into RedCAP and administered anonymously online (26, 27). Results were analyzed with descriptive statistics.

### Key informant interviews

Two co-authors (KBB, AAW) with extensive interview experience conducted 20–30 min interviews with 25 key informants between January to June 2023. Interviews were conducted over Zoom and audio-recorded, then transcribed. All interviews followed a pilot-tested interview guide with 5 open-ended questions (Supplementary Material). These focused on general impressions, how the VCA could be incorporated into existing curriculum for teaching and assessing error disclosure, and how these assessments might be helpful for resident evaluation and/or feedback. No field notes were made, and transcripts were not reviewed by participants.

### Interview content analysis

One co-author (EJG) reviewed a subset of six anonymized transcripts to develop an initial codebook. Questions from the interview guide framed initial codes, and subcodes were developed inductively. Two research associates (TM, KH), both trained in qualitative methods, reviewed five transcripts (including those used for initial codebook development and three new ones) to verify codebook comprehensiveness, confirm that key concepts and information needed for the study's objectives were identified and appropriately represented, and identify any additional emergent themes. They then refined the codebook in consultation with the original code book developers. To enhance trustworthiness, TM and KH independently coded a subset of five transcripts (20% of the total), met to reconcile discrepancies, and resolved outstanding issues through discussion with the broader team. Reflexivity was maintained through regular coder meetings and discussions of potential bias and interpretation. The team monitored for thematic saturation throughout the coding process, adding codes if additional themes emerged. Coding was performed using the John Snow NLP Lab (http://www.johnsnowlabs.com). The transcripts were coded manually without using NLP features in the coding software.

### Ethics

The University of Washington IRB approved all study procedures (Study 00015707). Participants provided verbal consent at the start of the interview and could opt out at any time.

## Results

### Participant characteristics

Of 40 faculty members invited to participate, 29 completed the VCA exercise, and 25 completed every step, a 63% completion rate that is expected for busy clinicians. All 7 programs were represented, with a median of 4 faculty participants per site (range 1–5). Table 1 displays details of the 25 participants' demographic characteristics and prior experience with supervising and teaching error disclosure. All faculty reported previously communicating with patients about harm in care they had provided but had variable experience with coaching residents in error disclosure (Table 1).

**Table 1:** Faculty participant demographics.

Faculty characteristic	Number (%)
Which gender do you identify with?	
Female	11 (44%)
Male	14 (56%)
Which race/ethnicity do you identify with?	
White	19 (76%)
Asian	5 (20%)
Latino/Hispanic	1 (4%)
How many years have you been in practice?	
0–5 years	4 (16%)
6–15 years	13 (52%)
16–25 years	6 (24%)
26+ years	2 (8%)
What is your specialty	
Internal Medicine	23 (92%)
Family Medicine	2 (8%)
Training site type	
Community-based	12 (48%)
University-based	13 (52%)
What is your job role in GME?	
Core Faculty	9 (36%)
Asst/Assoc Program Director	8 (32%)
Other GME/QI leader	5 (20%)
Program Director	3 (12%)
Have you previously coached a resident to disclose a harmful error?	
Yes	17 (68%)
No	4 (16%)
Not sure	4 (16%)
Have you previously observed a resident disclose a harmful error?	
Yes	16 (64%)
No	6 (24%)
Not sure	3 (12%)
Have you previously provided feedback to a resident about their disclosure of a harmful error?	
Yes	14 (56%)
No	9 (36%)
Not sure	2 (8%)
Have you previously taught error disclosure skills in other settings?	
Lecture	10 (40%)
Role play	5 (20%)
Standardized patient	4 (16%)

### AAF survey

Faculty rated the VCA with a mean AAF score of 4.23 (SD 0.78), corresponding to high acceptability with a rating of 5 as “completely acceptable”, although slight variation existed across domains (Table 2).

**Table 2:** Acceptability, appropriateness, and feasibility scale.

Acceptability of intervention measure (AIM)	Mean (SD)
The VCA program meets my approval.	4.24 (.88)
The VCA program is appealing to me.	4.20 (.91)
I like the VCA program.	4.12 (0.88)
I welcome the VCA program.	4.28 (0.79)
Total	4.21 (0.86) Range 2–5
Intervention appropriateness measure (IAM)	
The VCA program seems fitting.	4.12 (0.83)
The VCA program seems suitable.	4.25 (0.85)
The VCA program seems applicable.	4.32 (0.69)
The VCA program seems like a good match.	4.2 (0.76)
Total	4.22 (0.78) Range 2–5
Feasibility of intervention measure (FIM)	
The VCA program seems implementable.	4.32 (0.69)
The VCA program seems possible.	4.36 (0.70)
The VCA program seems doable.	4.32 (0.56)
The VCA program seems easy to use.	4.04 (0.89)
Total	4.26 (0.72) Range 2–5
Overall Average of AIM, IAM, FIM	4.23 (0.78)

### Key informant interviews

Faculty respondents identified several strengths and weaknesses of the VCA for error disclosure simulation. Key strengths included the VCA case quality and the tool's ability to fill a void in their current patient safety curriculum (Table 3). For example, most participants (19/25) commented that the cases were either relevant to their specialty or that they had encountered similar scenarios in their clinical work. One commented: “*Almost all of those things, I’m sure have happened or will happen. I’ve experienced, almost, every one of the adverse events that was described*.” Similarly, 15 of 25 faculty described the VCA actors and scripts as realistic, simulating the feeling of being in a clinical environment. Respondents noted that error disclosure was not a major part of their current curricula and that trainees should have more exposure to this content. One mentioned “*We probably should have been doing this sooner, quite honestly*.” The main limitation voiced by respondents (10/25) was the software's inability to create dynamic patient responses based upon the user's response, which contrasts to a standardized patient simulation with more fluid conversational exchanges.

**Table 3:** Key themes and quotations about VCA content, structure, software, and function.

Theme	Examples
Realistic content Description: The content included in the VCA cases felt realistic, simulating real-life emotional responses.	*“It simulated that feeling you get when patients confront you.”* *“[These scenarios] could have happened and were very realistic.”* *“It didn't feel artificial. It felt like something that could happen in real life.”* *“I thought true to life, and it was impressive in terms of its emotional reach for me.”*
Relevance Description: The content of the VCA cases were relevant to clinical practice.	*“The cases were very like appropriate and very real world; this is something I feel like I would have to face as a physician having to explain some of the errors.”*
Addressing user needs Description: Identified gap in current curriculum wherein VCA tool would be beneficial.	*“It's just like I'm grateful for this opportunity to insert this into our curriculum, and I'm thinking [to] myself, I hope we can do it in future years.”*
Ability to practice in a simulation Description: Discussion of the value of using interactive practice to try and improve communication skills.	*“I really liked that you were forced to come up with something to say to actually practice saying something out loud.”* *“Actually saying the word is very different than reading what might be a good response, or hearing someone else say what would be a good response.”* *“It did a good job of giving me an opportunity for more of a roleplay environment without needing someone else and perhaps with a standardized someone else.”* *“It's a really good, safe way, like a psychologically safe way to get some experience in doing that, make the mistakes, and get feedback on them.”*
Limitations Description: Discussed limitations of the VCA software.	*“You can't engage with that in the same way that you do an actual live person.”* *“It's not like it's very easy to just pick up standardized patients and ship them around the world or whatever so I would imagine a program or reproducible program would be limited to video records and vignettes and things like that, so which could still be effective, but obviously it would be a step down from having, you know, more role play and things like that live role play.”*
Need to create a psychologically safe framework for resident use Description: Discussion of how trainees benefit from psychologically safe learning environment	*“You get better participation by residents if they feel like it is completely low stakes…that nobody from the program or an evaluative standpoint can see [the results]”* *“I think this idea of being recorded bothers people enough that I would try to design [the curriculum] so it wasn't evaluative.”*

Respondents agreed that repeated cycles of practice and feedback during residency would be ideal, if protected curricular time could be found. All faculty stated that they would like residents to use the VCA at least annually, and 19 of 25 believed practice should occur more than once a year to maintain skills. The preferred timing during the academic year varied, based upon the specific program's course schedule or rotation calendar. Regarding barriers to implementation, nearly all faculty reported that the lack of dedicated time for this type of curriculum would be a challenge. Either asking residents to complete the cases on their own time, or finding a dedicated time and physical space during other didactic time were identified to be barriers. Despite the appeal of on-demand availability, the faculty were wary of asking trainees to complete an educational task without protecting time to do so. When residents requested to complete the VCA out of class, one respondent was “*kind of surprised at how many reminders I had to send*.” A few programs also expressed a lack of expert faculty available to present a lecture on error disclosure.

Faculty believed the VCA might supplement other assessments of communication skills, providing data about broader progress towards communication milestones. As one envisioned, repeated VCA use would allow residents to “*see their change over time…I think that'd be really meaningful to residents.*” Eighteen respondents felt the VCA would be a valuable remediation tool, including one respondent who said, “*For residents who have [a] challenge with patient communication, I think this could be one tool as a way to provide some sort of structured feedback*.” No consensus existed about whether the tool would be more appropriate for formative vs. summative feedback. For example, one respondent preferred a formative role in which “*Residents get to review their scores on their own, and it's sort of a self-driven development or learning process.*” However, others envisioned gathering summative data on residents, seeing a novel opportunity to measure skills and progress towards milestones: “*If we were actually, you know, acquiring summative data from this, it would be new. That said, I think particularly at a residency level, you want to demonstrate that people have competence in various domains, error disclosure being one of them*.”

Regarding whether faculty should review the resident's feedback, the majority of faculty (18/25) suggested that a faculty coach would be helpful to discuss the resident's feedback with them. Reasons for this recommendation included a belief that residents would take the task more seriously, coaching would augment learning, and a desire to support learners with the lowest performance. Yet, they worried faculty involvement could increase the sense that residents were being evaluated. Faculty recommended a coach with a comfortable relationship with the resident, “*not a program director*.” Faculty also felt the current ∼2-week delay between VCA practice and feedback would be a barrier to coaching. Multiple faculty members expressed that more rapid crowdsourced feedback would facilitate in-the-moment coaching.

Participants requested other software features to support curricular implementation, including a summary of an individual's scores over the course of residency, and real-time tracking of whether the trainees completed the VCA for use in class. Additionally, interviewees wanted to gauge the cohort's aggregate performance and determine whether there were any outliers in the program who might need more support or resources. For example, one program director said: “*I actually think the most important piece of that would be to identify outliers, especially on the low end.”*

## Discussion

This study explored the perceptions of residency faculty responsible for quality and safety training regarding the Video-based Communication Assessment (VCA) for teaching error disclosure communication skills. The VCA's acceptability, appropriateness, and feasibility measures were all high, suggesting the tool is well designed for the educational need. This aligns with outcomes of the related randomized trial, which demonstrated improvements in resident communication skills after using the VCA (24). Faculty appreciated the low-pressure nature of the VCA simulation environment and felt the cases were clinically relevant, realistic scenarios that fill a curricular void. Collectively, these findings indicate that the approach studied here (a lecture on error disclosure followed by brief rounds of VCA practice and feedback) is viable and appropriate for wider adoption to meet ACGME requirements and to prepare residents to engage with CRP processes.

Yet, faculty identified limitations of the VCA and barriers to adoption that highlight opportunities to refine the tool or provide anticipatory guidance. First, future users should plan carefully to protect curricular time for the VCA. Although the fact that the VCA can be used outside of class time would appear to be an advantage, faculty cautioned against creating burdens for residents outside of work hours. This matches the finding from the randomized trial that a significant minority of residents reported spending minimal time reviewing their VCA feedback outside of class. Second, some users wished the VCA could adapt to each user's response, making it more interactive. In response, software engineers might explore a role for artificial intelligence or branching logic in future conversational simulations; whether this can be done without losing assessment reliability is unknown. Third, respondents felt the VCA would be more useful if feedback were available more quickly. Future development efforts should explore techniques to expedite feedback without sacrificing accuracy and reliability.

Faculty identified several learning frameworks for the VCA, including potential roles in formative assessment, summative assessment, remediation, and coaching. Although most faculty believed it would be useful for remediation, it was not tested for that purpose in the accompanying educational trial, and new studies should be performed to assess its suitability for that purpose. Most participants also recommended providing a faculty “coach” or mentor, who would debrief results with residents. Faculty debriefing of resident error disclosure to a standardized patient did not improve self-efficacy in one prior study (28), but the widespread faculty interest in this approach supports studying coaching in the context of VCA-based training. Evidence of efficacy of the coaching model is needed before adoption.

Opinions regarding whether the tool should be used for more formative vs. summative assessment varied among programs and appeared to reflect a tension between programs' obligation to assure competency and the need to create a supportive learning environment. While summative VCA evaluations allow programs to better organize competency-based education, a greater emphasis on assessment appears to entail the tradeoff of eroding resident comfort and participation with the VCA. However program directors decide to review and use VCA scores, they should consider taking steps to clearly explain their approach and rationale to residents. A better understanding of resident viewpoints and VCA experiences would also help to inform deployment decisions.

This work has limitations, as the study has a modest sample size and may not be representative of residencies outside internal and family medicine. The participating residencies did not have robust error disclosure curricula prior to joining this study, and this work does not capture the viewpoints of faculty from programs which have existing simulation-based training in place. Not all transcripts were double-coded, which may have constrained the assessment of coding reliability across the entire dataset. We did not return transcripts to participants or perform member checking. The respondents were not provided with cost information about the tool nor asked to consider costs, which would also affect adoption. The VCA is not yet available for widespread use outside of research trials. Finally, this work does not address the viewpoints of resident users about using the VCA to develop and demonstrate competence at error disclosure.

## Conclusion

Faculty familiar with the VCA report that it is acceptable, appropriate, and feasible for teaching error disclosure communication skills in IM and FM residencies but testing with larger groups and evaluation in other specialties is warranted. Key opportunities to improve the system relate to simulation interactivity and feedback speed. To facilitate adoption, faculty recommended protecting curricular time for VCA use and effectively communicating with residents about who will review their personal assessments and how the exercise will support their learning.

## Data Availability

The raw data supporting the conclusions of this article will be made available by the authors, without undue reservation.
