# Supplementary material for: Incorporating the video communication assessment for error disclosure in residency curricula: a mixed methods study of faculty perceptions
Source: Front Health Serv. 2025 Aug 29;5:1503922. doi: 10.3389/frhs.2025.1503922 (PMC12425960; doi:10.3389/frhs.2025.1503922)
Supplement: Supplementary file 1 [file Table1.docx]

Supplementary Material: Interview Guide

**Interview Guide for Faculty Participants**

*Note: This script and set of instructions are intended as a guide for the interviewer. Interviewer discretion in phrasing, using probes and additional questions or explanation may be necessary because this is a qualitative interview. Qualitative interviews necessarily have a conversational aspect, and will almost always diverge at some points from a script. The interviewer may therefore adjust wording, for instance, to acknowledge and take into account that an interviewee has already offered some information in response to a prior question, to clarify a response, or to solicit more information.*

1. What did you think of the VCA overall? (what are its strengths and weaknesses?)
2. We’re interested to learn how a residency program like yours might make the VCA part of its longitudinal curriculum for teaching and assessing error disclosure.
   1. What frequency of practice would be ideal (eg all at once, or spread out with a case every 6 months)?
   2. Who should review the results? (e.g. resident only vs include a faculty member or program director)?
   3. What logistical and practical barriers do you anticipate to implementing the VCA program?
3. We are considering developing a web-based interface that allows program directors to review the assessments of residents who have used the VCA. What features or information in a web-based program director interface would be important to you? (would you want to hear responses or just scores, for example)
4. How might the VCA integrate with other assessments of resident communication tools?
   1. Could you envision using the VCA as a remediation tool to provide additional practice for selected residents?
5. We welcome your feedback on the VCA overall – are there any comments you would like to share?
